# Cord blood in regenerative medicine: do we need immune suppression?

**DOI:** 10.1186/1479-5876-5-8

**Published:** 2007-01-30

**Authors:** Neil H Riordan, Kyle Chan, Annette M Marleau, Thomas E Ichim

**Affiliations:** 1 Medistem Laboratories Inc, Tempe Arizona, USA; 2 Institute for Molecular Medicine, Huntington Beach, California, USA

## Abstract

Cord blood is currently used as an alternative to bone marrow as a source of stem cells for hematopoietic reconstitution after ablation. It is also under intense preclinical investigation for a variety of indications ranging from stroke, to limb ischemia, to myocardial regeneration. A major drawback in the current use of cord blood is that substantial morbidity and mortality are associated with pre-transplant ablation of the recipient hematopoietic system. Here we raise the possibility that due to unique immunological properties of both the stem cell and non-stem cell components of cord blood, it may be possible to utilize allogeneic cells for regenerative applications without needing to fully compromise the recipient immune system. Issues raised will include: graft versus host potential, the immunogeneicity of the cord blood graft, and the parallels between cord blood transplantation and fetal to maternal trafficking. The previous use of unmatched cord blood in absence of any immune ablation, as well as potential steps for widespread clinical implementation of allogeneic cord blood grafts will also be discussed.

## Background

The first widespread utilization of cord blood as a stem cell source was in the treatment of pediatric hematological malignancies after myeloablative conditioning. Since matching requirements for this type of transplant are not as strict as for hematopoietic stem cell sources, cord blood began gaining acceptance in adult patients lacking bone marrow donors [1-6]. Outside the area of oncology, the clinical use of cord blood has expanded into various areas that range from reconstituting a defective immune system [7], to correcting congenital hematological abnormalities [8], to inducing angiogenesis [9]. A sample of some of cord blood clinical studies addressing non-malignant disorders is presented in Table 1.

**Table 1:** Non-Malignant Disorders Treated With Cord Blood

**Disorder**	**Number Treated**	**Outcome**	**Ref.**
Hurler's syndrome	20	17 of the 20 children were alive a median of 905 days after transplantation, with complete donor chimerism and normal peripheral-blood alpha-L-iduronidase activity	[100]
Duchenne muscular dystrophy	1	On 42nd day, physical examination revealed obviously improvement in walking, turning the body over, and standing up	[101]
Malignant infantile osteopetrosis	1	Normalization of spine bone mineral density.	[102]
Rothmund-Thomson syndrome	1	Complete immune reconstitution	[55]
Buerger's disease	4	Ischemic rest pain suddenly disappeared. Digital capillaries were increased in number and size.	[84]
Spinal Cord Injury	1	Improved sensory perception and movement in the SPI patient's hips and thighs within 41 days of cell transplantation. Regeneration of the spinal cord at the injured site	[85]
Krabbe's disease	25	Progressive central myelination and continued gains in developmental skills, and most had age-appropriate cognitive function and receptive language skills in patient subset	[14]
Omenn syndrome	1	T cell reconstitution	[103]
Non-healing wounds	2	Accelerated healing	[86]
Refractory anemia	3	All patients are alive and free of disease at between 17 and 39 months after cord blood administration	[104]
Diamond-Blackfan anemia	1	Successful seroconversion to vaccines (diphtheria, pertussis, tetanus, rubella, measles, and BCG) administered 22–34 months post-transplant.	[105]
Severe chronic active Epstein-Barr virus	1	Complete remission without circulating EBV-DNA has continued for 15 months transplant.	[106]
Behcet's disease	1	Twenty-three months after CBT, the patient is doing well and has no signs or symptoms of Behcet's disease	[9]
Mucopolysaccharidosis type IIB (Hunter syndrome)	1	Two years after transplant approximately 55% normal plasma iduronate sulfatase. activity has been restored and abnormal urinary excretion of glycosaminoglycans has nearly completely resolved.	[107]

In addition to current clinical use, cord blood is currently under intense experimental investigation in preclinical models of pathophysiologies that range from myocardial ischemia, to stroke, to muscle regeneration [10-13]. It is anticipated that in the next several years that widespread clinical entry of cord blood for non-hematopoietic tissue regeneration will occur. When this happens, the main question will be how to select patients that can be myeloablated so as to allow acceptance of the cord blood graft. According to current dogma in the discipline, it is believed that myeloablation, or at minimum non-ablative immune suppression of the recipient is strictly required. In situations of hematological malignancy it is desirable to myeloablate the recipient so as to eradicate the leukemic population while creating "space" for the donor cells to engraft. However, the question is, in patients that are not suffering from a disease that is associated with an aberrant bone marrow such as hematological malignancies or immunological dysfunctions, how is it justifiable to subject them to the high levels of morbidity and mortality associated with immune suppression? For conditions such as Krabbe disease where patients rarely survive beyond the age of 2 and cord blood transplant was demonstrated to induce 100% survival in a subgroup of patients treated [14], the justification for myeloablation can be made. However for conditions such as post-stroke regeneration or induction of angiogenesis in angina patients, in which the population already suffers from major comorbidities and the potential benefit of cord blood therapy is only speculative, the ability to justify myeloablative protocols rapidly diminishes.

The purpose of this paper is to put forth the notion that the immunology of cord blood transplants for regenerative applications has to be viewed differently from the perspective and the practice of cord blood transplants for hematopoietic reconstitution. Specifically, we will provide reasons and rational for why in some situations, administration of cord blood, or stem cells derived thereof, may be possible with no, or minimal immune suppression of the recipient. Evaluation of this possibility will lead to acceleration of clinical entry and wide-spread utilization of cord blood transplants for non-hematopoietic indications.

## Regenerative cells in cord blood

Numerous publications have described the regenerative ability of cord blood cells in a myriad of preclinical disease models. Although the purpose of this paper is to discuss the immunology of cord blood transplants for regenerative uses, we will first overview some of the therapeutic stem cell populations found in cord blood so that we may discuss their immunogenic consequences later in the paper.

### Hematopoietic stem cells

The original clinically attractive feature of cord blood was the high concentration of hematopoietic stem cells, which is similar to that found in bone marrow: approximately 0.1–0.8 CD34^+ ^cells per 100 nucleated cells. However, in contrast to marrow, CD34^+ ^cells from cord blood possess higher proliferative potential in vitro [15], superior numbers of long term culture initiating cells and SCID repopulating cells [16,17], as well as higher telomerase activity [18]. The potent hematopoietic activity of cord blood derived CD34^+ ^cells may be attributed to the fact that cord blood is a much more developmentally immature source of stem cells as opposed to stem cells derived from adult sources. Attesting to the robust hematopoietic activity of cord blood derived CD34^+ ^cells in comparison to bone marrow cells is the fact that successful reconstitution, albeit delayed, of post-ablative hematopoiesis occurs in patients receiving approximately one tenth of the total nucleated cell number in a cord blood graft compared to a bone marrow graft.

### Endothelial progenitors and angiogenesis stimulating cells

In addition to being a source of hematopoietic cells, cord blood contains potent angiogenesis stimulating cells. Several phenotypes have been ascribed to cord blood angiogenic stimulating cells. In one report, the CD34^+^, CD11b^+ ^fraction, which is approximately less than half of the CD34^+ ^fraction of cord blood was demonstrated to possess ability to differentiate into functional endothelial cells in vitro and in vivo [19]. In another report, VEGF-R3^+^, CD34^+ ^cells were shown to possess not only the ability to differentiate into endothelial cells in vivo, but also to be able to expand approximately 40-fold in vitro and subsequently maintain angiogenic function in vivo. The same study demonstrated that the concentration of this endothelial progenitor fraction found in cord blood CD34^+ ^cells is approximately tenfold higher as compared to bone marrow CD34^+ ^cells [20]. Regardless of the phenotype of the cord blood cell with angiogenesis stimulating ability, unfractionated cord blood mononuclear cells have also been used in numerous animal models [21-23], as well as in the clinic [9], for successful stimulation of angiogenesis.

In addition to endothelial progenitors, mesenchymal stem cells (discussed below in more detail), which are found in cord blood, are known to secrete numerous cytokines and growth factors such as VEGF and FGF-2 [24,25] which stimulate angiogenic processes. In fact, there are reports of mesenchymal stem cells contributing to angiogenesis through direct differentiation into endothelial cells [26].

### Mesenchymal stem cells

Mesenchymal stem cells are a type of cell capable of differentiating into various non-hematopoietic tissues. Currently this cell population is second to bone marrow stem cells in terms of clinical entry in that Phase III clinical trials are already underway with these cells. Mesenchymal stem cells are classically defined as adhere to plastic and expressing a non-hematopoietic cell surface phenotype, consisting of CD34^-^, CD45^-^, HLA-DR^-^, while possessing markers such as STRO-1, VCAM, CD13, CD29, CD44, CD90, CD105, SH-3, and STRO-1 [27]. To date mesenchymal stem cells have been purified from bone marrow [28], adipose tissue [29], placenta [30,31], scalp tissue [32] and cord blood [33]. Cord blood-derived mesenchymal stem cells have demonstrated ability to differentiate into a wide variety of tissues in vitro including neuronal [34-36], hepatic [37,38], osteoblastic [39], and cardiac [33]. An important aspect of this cell population is their anti-inflammatory and immunomodulator activity. For example, they constitutively secrete immune inhibitory cytokines such as IL-10 and TGF-β while maintaining ability to present antigens to T cells, thus suggesting they may act as a tolerogenic antigen presenting cell [40,41]. Conceptually, the mesenchymal content of umbilical cord blood grafts may explain the tolerogenic capabilities, which some have speculated to be donor specific.

Although the majority of published studies have examined bone marrow derived mesenchymal stem cells, and thus are outside the scope of the present review, it is important to note differences between mesenchymal stem cells derived from different sources. A recent study compared mesenchymal stem cells from bone marrow, cord blood and adipose. Cord blood mesenchymal stem cells which were capable of expansion to approximately 20 times, whereas adipose derived cells expanded an average of 8 times and bone marrow derived cells expanded 5 times [42]. This, and other studies support the important role of mesenchymal stem cell content in the biological activities of the cord blood graft.

### Unrestricted somatic stem cells

Cells with markers and activities resembling embryonic stem cells have been found in cord blood. Zhao et al identified a population of CD34^- ^cells expressing OCT-4, Nanog, SSEA-3 and SSEA-4, which could differentiate into cells of the mesoderm, ectoderm and endoderm lineage. In vivo administration of these cells into the streptozotocin-induced murine model of diabetes was able to significantly reduce hypoglycemia [43]. The existence of cells with such pluripotency in cord blood was also observed by Kogler et al who identified an Unrestricted Somatic Stem Cell (USSC) with capability of differentiation into functional osteoblasts, chondroblasts, adipocytes, hematopoietic and neural cells. USSC were demonstrated to be capable of > 40 population doublings in vitro without spontaneous differentiation or loss of telomere length. Interestingly, administration of these cells (derived from human cord blood) into fetal sheep resulted significant human hematopoiesis (up to 5%), hepatic chimerism with > 20% albumin-producing human parenchymal hepatic cells, as well as detection of human cardiomyocytes. The mechanism of differentiation was not associated with fusion [44]. Support for presence of such pluripotency in cord blood cells also comes from a similar experiment in which CD34^+ ^Lineage^- ^cells were transfected with GFP and administered *in utero *to goats. GFP^+ ^cells were detected in blood, bone marrow, spleen, liver, kidney, muscle, lung, and heart of the recipient goats (1.2–36% of all cells examined) [45]. In other studies, McGuckin et al demonstrated that culture of cord blood cells that have been depleted of lineage committed cells in a TPO, c-kit, and flt-3 ligand culture express markers of embryonic stem cells including TRA-1–60, TRA-1–81, SSEA-4, SSEA-3 and Oct-4. Functionally, these cells also demonstrated pluripotent differentiation ability and in vivo hematopoietic activity [46-48].

## Cord blood transplantation without host preconditioning: will there be GVHD?

The possibility of using cord blood in absence of host preconditioning would open up the door for a multitude of stem cell therapeutic applications. The currently dogma amongst cord blood transplanters is that administration of allogeneic cord blood, even if HLA-matched, would in the best case scenario lead to immunologically-mediated rejection or the graft, and in the worst case cause GVHD. Here we provide rationale for the preliminary clinical exploration of cord blood administration in a non-preconditioned host.

### ABO-matched, HLA-mismatched transfusions

In the 1930s it was reported that cord blood could be safely used as a substitute for peripheral blood for performing transfusions [49]. Since HLA-matching was not available at that time and no adverse effects were noted, the feasibility of cord blood administration to a non-preconditioned host was suggested. A more recent Lancet publication described the use of cord blood as a source of blood donation for malaria infested regions in Africa. 128 pediatric patients with severe anemia needing transfusions were transplanted with an average of 85 ml of ABO matched cord blood with no HLA matching. No report of graft versus host was noted, and cord blood was proposed as a transfusion source when peripheral blood is not available due to economical or social reasons [50]. An extensive review of 129 patients transplanted with a total of 413 Units of cord blood (average 86 ml) with no preconditioning or HLA matching between 1999 to 2004 was published by Bhattacharya [51]. Of these patients, aged 2–86 years old and suffering from advanced cancer (56.58%) and other diseases (43.42%) such as ankylosing spondylitis, lupus erythematosus, rheumatoid arthritis, aplastic anemia, and thalassemia major, no immunological reactions were noted in patients followed for 1–4 years. The same author reported other patient cohorts that have been similarly treated and had no GVHD or other immune reactions [52-55]. Furthermore, transfusion of cord blood in non-HLA matched recipients was also associated with transient increases in peripheral CD34 counts, without evidence of GVHD in patients with cancer and HIV [56,57]. Unfortunately in these studies did not perform long-term molecular analysis for chimerism. Despite this drawback, it is evident from the initial work in the 1930s, to the numerous cases reported by Bhattacharya, to the publication in Lancet, that administration of cord blood is a safe procedure not associated with immunological consequences. Thus based on the current data, the worse a cord blood transplant will do is do nothing.

### Administration of adult lymphocytes does not elicit GVHD

The cells that are "dangerous" from the cord blood from a GVHD perspective are lymphocytes that may have alloreactive potential. Lymphocytes from cord blood, in contrast to adult blood, are generally immature and usually do not secrete as many inflammatory cytokines. Therefore administration of allogeneic lymphocytes purified from adult blood would be a much more dangerous procedure, at least as far as GVHD is concerned, in contrast to administration of lymphocytes from cord blood. The fact is administration of lymphocytes from paternal sources has been performed in numerous reports in the clinical practice of using "paternal lymphocyte immunotherapy" for treatment of spontaneous abortions. Numerous trials have been conducted administering doses of up to 2 × 10^9 ^paternal lymphocytes into pregnant mothers who have had recurrent miscarriages [58,59]. These doses are higher than the 1.5–3 × 10^7 ^nucleated cells/kg administered during a cord blood transplant [60]. Interestingly, in pregnant women administered these high doses of completely allogeneic cells, no GVHD has ever been observed, although Th2 immune deviation has been reported by some groups [61,62]. Thus according to the current evidence, there is no fear of GVHD being induced after cord blood transplant. Bhattacharya even administered as many as 32 units of cord blood to an individual without seeing GVHD [57].

### Homeostatic proliferation in lymphopenic environment causes GVHD

The reader will ask, in response to the above arguments regarding GVHD-inducing ability of cord blood, "*why is GVHD, a clinical reality in patients receiving cord blood for hematological malignancies?" *The answer is that current day cord blood transplants take place following ablation of host T cells. This creation of an "empty compartment" allows for homeostatic expansion of the newly introduced T cells, which primes them for aggressive immune reactions and alleviates their requirement for costimulation [63]. It is known from the transplantation literature that T cells reconstituting a host that has been lymphoablated are resistant to costimulatory blockade and tolerance induction [64]. Furthermore, the pioneering experiments of Rosenberg's group demonstrated that infusion of tumor specific lymphocytes following ablation of the recipient T cells, using conditions similar to those used in cord blood transplant preconditioning allows for highly aggressive anti-tumor responses that otherwise would not be observed [65]. Further supporting the concept that reconstitution of a lymphocyte deficient immune system can cause immune hyperreactivity comes from clinical observations of "autologous GVHD" in patients administered drugs associated with induction of lymphopenia [66,67]. We therefore propose that GVHD is not an intrinsic property of the allogeneic cells introduced into the host, but a result of the lymphoablation induced in the recipient prior to cellular administration.

## Will the graft be cleared?

If cord blood can be administered into a non-preconditioned patient without fear of GVHD, then the next question arises as to whether the infused cells will actually endow some type of benefit or be rapidly cleared by the immune system. As previously mentioned, biological effects of mismatched cells, even if they are cleared by the immune system may have a beneficial role in inflammatory pathologies through exertion of a Th2 phenotype as seen in mothers being administered their mate's lymphocytes. Furthermore, even if transplanted cells are cleared by the immune system, it is known that apoptotic cells can mediate various therapeutic anti-inflammatory effects that are clinically relevant [68]. Animal models suggest that human cord blood cells may be administered for therapeutic benefit into other species, in absence of immune suppression. For example, Vendrame et al reported that administration of human cord blood into a non-immunosuppressed rat model of stroke resulted in increased neuronal survival, and interestingly decreased inflammatory infiltrates as compared to controls [69].

### Fetal cells do not get cleared by maternal immune system

However, we do not believe that complete immune mediated clearing of regeneratively-important cord blood constituents occurs in the allogeneic setting. One reason for this notion comes from an interesting phenomena observed in pregnancy. It is well established that during pregnancy fetal cells enter maternal circulation [70]. While circulating CD34^+ ^cells of fetal origin are found a percentage of women who have had children [71], in the bone marrow 100% of women who have had children were found to contain offspring-derived mesenchymal cells in their bone marrow [72]. Although some studies have correlated autoimmunity with residual lymphocytes causing a GVHD-like reaction in the mother, more careful analysis of these studies show that immune cells of fetal origin are largely outnumbered by cells of maternal origin. This is the basis for the proposition of Khosrotehrani et al that the fetal cells are actually "pregnancy associated progenitor cells" that act as allogeneic "repair cells" [73]. The authors of this hypothesis believe that these repair cells are actually migrating to the site of autoimmune damage in order to control injury and cause regeneration. The authors cite numerous examples in support of their idea, more notably, a case report of a hepatitis C patient who stopped treatment but disease relapse was not observed. Biopsy analysis demonstrated the liver parenchyma was heavily populated with cells of male origin that based on DNA polymorphism analysis were derived from a previous pregnancy more than a decade earlier [74]. Additionally, they cite reports of fetal cells differentiating into thyroid, cervix, gallbladder and intestinal epithelial cells [75-78]. Data from animal models, although scarce, supports the notion that fetal cells trafficking into the mother may play some reparative function. For example, it was reported that EGFP expressing fetal cells would selectively home into damaged maternal renal and hepatic tissues after gentamycin and ethanol induced injury [79]. Furthermore, another study demonstrated that subsequent to excitotoxic injury in the maternal brain, fetal-derived EFGP positive cells can be identified which express morphology and markers of neurons, astrocytes, and oligodendrocytes [80]. The authors of this paper are not stating that the fetal transfer of mesenchymal cells to the maternal host is an exact duplicate of an allogeneic cord blood transplant in absence of immune suppression. Rather, we are proposing fetal to maternal trafficking as a possible example of a natural biological situation in which stem cells may persist in an allogeneic environment without induction of complete tolerance.

### Mesenchymal stem cells do not need myeloablation for efficacy

Currently there are several ongoing clinical trials in Phase I-III using "universal donor" mesenchymal stem cells in non-conditioned recipients for treatment of Crohn's disease [81], GVHD [82], and myocardial infarction [83]. Although these cells are bone marrow expanded mesenchymal cells, the superior proliferative potential of cord blood mesenchymal cells may allow them to not only escape immune destruction, but also expand in vivo and mediate therapeutic effects superior to those derived from the bone marrow. The fact that regulatory agencies have allowed advancement of "off-the-shelf" universal donor mesenchymal stem cells supports the numerous reports of clinical efficacy in an allogeneic setting.

### Clinical evidence of cord blood efficacy in absence of myeloablation

To the knowledge of the authors, there have only been 3 published reports of non-conditioned recipients receiving cord blood cells for regenerative purposes. The first report is of 4 patients with Buerger's disease who were administered 4/6 HLA matched allogeneic cord blood cells locally in the area of limb ischemia. In all patients rest pain disappeared and necrotic lesions healed approximately 4 weeks subsequent to cell administration. Significant angiographically evidenced neoangiogenesis proximal to area of administration was observed [84]. No graft versus host, or inflammation was observed at the site of injection. The second published report is of a matrix delivered allogeneic cord blood dose to a patient who suffered from spinal cord injury. Improved sensory perception and movement was observed, as well as CT and MRI observation of tissue regeneration at site of injury was reported [85]. The third report described 2 patients with non-healing wounds who were treated with autologous fibrin glue containing matched (2 mismatches allowed) allogeneic cord blood isolated CD34^+ ^cells. Significant wound healing was observed with no indication of GVHD at 3 and 7 months subsequent to treatment [86]. These reports give some suggestion that administration of mismatched cord blood stem cells may endow potentially therapeutic benefit without rejection, at least immediate rejection, by the host versus graft process. More importantly, these studies demonstrate that allogeneic cord blood cells may be used clinically without immune-mediated clearing before therapeutic properties are exerted.

## Strategies for clinical implementation

Given the unique regenerative capabilities of cord blood, the easy accessibility of HLA matched donors, and relative inexpensiveness as compared to other cellular therapies; it is of great interest therapeutically to expand its use into non-conditioned recipients. Below we will provide some initial thoughts on how this may be performed.

### Cord blood together with stem cell activators

One simple method of stem cell therapy would be administration of cord blood units in patients with degenerative diseases in the form of direct transfusions has described by Bhattacharya [57]. This approach, however has not demonstrated significant regenerative activity. A more promising approach may be administration of cord blood cells in combination with activators of endogenous stem cells. For example, clinically used agents such as thalidomide [87], valproic acid [88], or 5-azacytidine [89,90] all have demonstrated ability to induce proliferation of CD34^+ ^stem cells in vitro and/or in vivo. In the majority of cases, stem cell activating compounds are administered for therapeutic benefit in absence of addition of exogenous stem cell sources. One example is current clinical trials of hCG and EPO for treatment of acute stroke [91]. The addition of exogeneous allogeneic stem cells to clinically accepted stem cell expanding agents may be a relatively easy initial clinical trial of regenerative cord blood therapy in absence of recipient conditioning. An interesting addition to administration of stem cell activators is the localized introduction of stem cell specific chemoattractant agents at the site in need of repair, followed by systemic administration of cord blood stem cells. Chemoattractant agents could include stromal derived growth factor-1 [92], other various agonists of CXCR-4 [93], or hepatocyte growth factor [94].

### Cord blood plus natural chemoattractant

An alternative approach to administration of exogenous stem cell chemoattractants would be to administer stem cells at the narrow window period after tissue injury when endogenous chemoattractants are secreted by the injured tissue. For example, following myocardial infarction, as well as stroke, there is a period of time which concentration of local stem cell chemoattractants are so high that bone marrow derived progenitors are actually mobilized into systemic circulation [95]. Activators of endogenous stem cells may be administered to allow localized tissue repair, while exogenous stem cells are administered to provide support to the activated endogenous cells.

### De-immunogenized cord blood graft

Clinical entry of a cord blood therapeutic in patients who are not preconditioned would require a high margin of safety to be met. Accordingly, one approach for beginning cord blood clinical trials may be administration of grafts that are depleted of T cells, B cells, and dendritic cells. In this manner, even the remote possibility of GVHD would be negated, and the there would be no B cell or dendritic cell induced antigenicity. Essentially, the percentage stem cell content of the graft would increase while the possible immunogenic components would decrease. A method of accomplishing this would be the pretreatment of cord blood units with the clinically used anti-CD52 monoclonal antibody CAMPATH. It has been previously demonstrated that this agent can be used in substantially "cleaning" grafts of T cells without affecting hematopoietic activity both in vitro [96] and in the clinic [97]. Furthermore, CAMPATH has been shown to deplete B cells [97], as well as circulating blood dendritic cells [98,99]. An ideal indication for utilization of such a treatment would be in limb ischemia in which a variety of stem cell sources have demonstrated angiogenic effects, however clinical implementation of stem cells for limb ischemia is currently limited by the difficulties of obtaining and purifying autologous stem cells.

## Conclusion

In summary, the authors propose that expanding the use of cord blood to non-preconditioned adult recipients for regenerative purposes would be a great step for the practical advancement of stem cell therapeutics. By overcoming allogeneic barriers in regenerative medicine, the fundamental limitations of autologous cell therapy may result in effective standardized "off-the-shelf" cellular products for regenerative therapeutics. This major step can only be performed by understanding the unique immunology of cord blood grafts, leveraging the graft's regenerative capability for specific indications, and identifying methods of amplifying cellular effects through administration of various drugs.
